# How weekly monitoring variables influence players’ and teams’ match performance in elite futsal players

**DOI:** 10.5114/biolsport.2023.112091

**Published:** 2022-01-03

**Authors:** João Nuno Ribeiro, Diogo Monteiro, Jaime Sampaio, Micael Couceiro, Bruno Travassos

**Affiliations:** 1 Department of Sport Sciences, University of Beira Interior, Covilhã, Portugal; 2 Research Centre in Sport Sciences, Health Sciences and Human Development, CIDESD, Vila Real Portugal; 3 ESECS, Polytechnic of Leiria, Leiria, Portugal; 4 University of Trás-os-Montes and Alto Douro, Vila Real, Portugal; 5 Ingeniarius, Lda., Alfena, Portugal; 6 Portugal Football School, Portuguese Football Federation, Oeiras, Portugal

**Keywords:** Monitoring system, Weekly training load, Readiness, Match outcome

## Abstract

This study aimed to investigate how weekly training load constrains the performance of players and teams in official futsal competitions. Data from a professional male team were collected during two seasons (46 weeks). The applied monitoring system analysed the training load (as measured by session perceived exertion, sRPE), the total recovery status (TQR), the well-being score (WBs) and the variability of neuromuscular performance during each week (CMJ-cv). In addition, the performance was assessed for all the matches. A path analysis model was performed to test the associations across variables. Results from the path analysis model revealed that it explains 31% of the teams’ performance. In general, the results show that previous team performance has no significant effects on the training week. A significant negative relationship was found between CMJ-cv and match performance (*β* = -.34; CI95% -.359 to -.070), as well as a significant negative relationship between players’ match performance and the team’s match performance (*β* = -.55; CI95% -.292 to .740). Regarding indirect effects, only a negative association between CMJ-cv and team match performance via players’ match performance (*β* = -.19; CI95% -.342 to -.049) was identified. The small variation of the weekly CMJ (CMJ-cv) seems to be a key variable to monitor and explain both player and team performance. Based on this model, and only looking at the physical variables, it was possible to explain 31% of the team’s performance. Longitudinal and multi-team studies should be conducted to integrate other technical, tactical and psychological variables that allow the level of understanding of players’ and teams’ performance to be improved.

## INTRODUCTION

In team sports, the main purpose of the training process is to choose and manage the stimulus that optimize the player/team performance for competition, i.e. allow the players to start the competition with high levels of fitness, motivation, cognitive capacities and a low level of fatigue [1].

In elite futsal, the competition phase usually runs for 9–10 months, with official matches played almost every week and sometimes twice a week [2]. For this purpose, it is important to define an athlete monitoring system that ensures a balance between training load (the product of type, volume and intensity of training), recovery status (individual athlete’s response to that load) and readiness for competition [1, 3, 4].

In recent years, the development of monitoring systems in sport has occurred due to the evolution and availability of wearable technologies. These technologies make it possible, for example, to track and register internal and external load or even the positional relations between players using the Global Positioning System for outdoor modalities or, more recently, ultra-wideband technology for indoor modalities [5–7].

However, for some athletes/teams/squads, insufficient resources can be a major reason for not developing and implementing a monitoring system [8]. Based on that, friendly and understandable common tools [8, 9], which include subjective well-being questionnaires (mood, stress, fatigue, soreness and sleep) (WBs) and their derivatives, such as session rated perceived exertion (sRPE) and the total quality recovery (TQR), have been frequently used in team sports [10].

In an attempt to build the relationship between training load and WBs, some authors revealed that the muscle soreness and fatigue measured with a well-being questionnaire were moderately and inversely correlated with sRPE [11]. In line with that, in another study, the authors found that higher TQR seems to be related to better self-reported sleep quality [12].

Despite the reliability and informative value of subjective measures of training load and well-being [13], these should be combined with more precise and objective variables to ensure a balance between athlete perception and actual performance capacity [4].

Vertical jump height is one of the most reliable measures to quantify the athletic performance and the training-related fatigue in elite players [14, 15].

However, more than analysing physical and wellness data in isolation, there is a need to contextualize the data for a clear understanding of the results [16]. For instance, opponent standard and match outcome seem to affect weekly training load values. When looking particularly to match outcomes in elite soccer, it was revealed that players perceived a higher training load after a defeat or a draw compared to a win [17, 18]. Despite the particularities of each sport, in elite Australian football teams, it was noted that a balance on training load during the week increases the chances of teams winning the match [19]. However, while this relationship is interesting, there is a paucity of research examining the weekly training-performance relationship, particularly in futsal. In fact, in opposition to previous research, coaches always try to integrate the analysis of weekly training load with match performance indicators to improve the ability to understand the relationship between the process and the result [16].

Match performance indicators can be defined as a selection and combination of variables (e.g., passes, shots, goals, dribbles, interceptions, tackles) that characterizes individual or team performance helping to explain the team’s match performance [20]. Studies in other team sports suggest that the match performance indicators are fundamental for coaches to adjust the systems and strategies of play to the next matches and to cogitate about next week’s programme [21]. Previous research has revealed high reliability of such variables and a relationship with match outcome and teams’ level [22] or even with match external load [23].

The aim of the current study was to investigate how weekly training load constrains players’ and teams’ match performance through the influence of previous team performance. It was expected to observe: i) an effect of previous team performance on weekly training load; ii) a negative relationship between session rated perceived exertion (sRPE), total quality recovery (TQR) and well-being score (WBs) with countermovement jump variability (CMJ-cv); iii) a negative effect of CMJ-cv on players’ and teams’ match performance.

## MATERIALS AND METHODS

### Procedures

An observational descriptive study was carried out during the competitive phase of 2018–2019 and 2019–2020 seasons. Data from 230 training sessions, and from 46 microcycles, were collected, with only one match per week and 5 days of training session prior to the match. The matches included 23 wins, 5 draws and 18 losses. The weekly training programme was planned entirely by the team’s coaching staff and aimed to develop an integrated content (i.e., tactical, technical and physical factors were amalgamated) throughout the microcycle, which was divided into 3 physical periodization goals: Recovery (MD-5 [i.e., 5 days before a match]), Acquisition (MD-4/ MD-3 [i.e., 4 and 3 days before a match]) and Tapering (MD-2/ MD-1 [i.e., 2 and 1 days before a match]). As recovery strategies, all players performed a 5-minute cold-water immersion every MD-4 and MD-2 throughout the in-season.

### Participants and Setting

Data were collected from one single team, comprising 19 professional male futsal players (age: 24.5 ± 3.8 years; height: 173.6 ± 5.4 cm; weight: 70.3 ± 7.6 kg) who participated in the Portuguese first futsal league (Liga Placard). Of the 15 players who started the 2018–19 season, 11 remained in 2019–20, and 5 new players were part of the new season.

For players to be included in the data analysis, they had to meet the following criteria: (i) have participated in more than 80% of the weekly training sessions, and (ii) have started each week with medical clearance to compete.

The experimental procedures used in this study were in accordance with the Declaration of Helsinki and were approved by the local Ethics and Scientific Committee of University of Beira Interior (CEUBI-Pj-2018-029). All players in the sample were familiar with the club’s standard monitoring routine. The club and players provided written informed consent to allow the use of data.

### Measures

The monitoring tools included the daily analysis of the training load through sRPE and the recovery status using the TQR scale. Hooper’s Index questionnaire was used to record WBs and CMJ was used as a physical performance test and fatigue control measure, both performed only twice a week (see Table 1). All these data were collected all training sessions over the two in-seasons. Player match performance and team match performance were collected from all the matches played over the two seasons.

**TABLE 1:** Tracking weekly training variables of athlete monitoring system

Match Day	MD-5		MD-4		MD-3		MD-2		MD-1		Game
Physical Intention	Recovery		Acquisition		Acquisition		Tapering		Tapering		Competition

Monitoring Tools	Before	After	Before	After	Before	After	Before	After	Before	After	After

TQR	**x**		**x**		**x**		**x**		**x**		
sRPE		**x**		**x**		**x**		**x**		**x**	
WBs			**x**				**x**				
CMJ			**x**				**x**				
PMP											**x**
TMP											**x**

TQR – Total Quality Recovery; s-RPE – Session Rated Perceived Exertion; WBs – Well-being Score; CMJ – Countermovement Jump; PMP – Player’s Match Performance; TMP – Team Match Performance

### Session Perceived Exertion

The training load was quantified by the sRPE method. This method has been validated for monitoring internal training load in futsal [24].

Thirty minutes after the end of each daily training session (6 pm – 7 pm) athletes were presented with 10-point RPE scale and answered the question, “How intense was the training session?” using a visual analogue scale in which 0 means “not at all” and 10 “maximum effort”. The sRPE was calculated by multiplying the reported RPE score and the total time of the session, in minutes, which represents the overall load of the session in terms of AU. Higher values of sRPE correspond to higher values of training load.

### State of Recovery

To assess the state of recovery, the players answered the TQR scale. Before the start of the training session, the athletes answered the question “How recovered do you feel?” on a scale of 6–20, with 6 meaning being rested and 20 meaning extremely good recovery. The weekly average TQR score for each athlete was calculated. Higher values of TQR represent good levels of recovery during the week.

### Well-being Score

Individual responses to training demands were measured by the well-being questionnaire which was administered every morning (9 am – 10 am) twice in the week (MD-4 and MD-2). Players completed a short questionnaire on their smartphone using a Google doc form. The questionnaire had 5 separate aspects of player well-being [25]. These were: 1) How sore do your muscles feel today? 2) How fatigued do you feel today? 3) How well did you sleep last night? 4) How is your mood today? 5) How stressed do you feel today? Each question was score using a 1–5 Likert scale with 1 representing a low score and 5 a high score. These responses were then converted into a global WBs (%).

### Neuromuscular Performance

In order to monitor fatigue and neuromuscular performance, jump height was measured during a CMJ test (Optojump; Microgate, Bolzano, Italy) performed twice a week: MD-4 and MD-2. The test was performed before the training session in a randomized order. A standard warm up programme was completed prior to the test consisting of 10 minutes on a stationary bike followed by dynamic stretching and 3 trial jumps with increased intensity. Finally, they performed 3 jumps with approximately 45–60 seconds recovery between them. All jumps were performed with the players in the tall standing position, with both feet placed hip to shoulder apart and hands akimbo. The mean value of the three attempts of CMJ in the two evaluation moments of the week was used to calculate the coefficient variation of the jump height (CMJ-cv). The CMJ-cv expresses the variations on athletic performance of players over the week, with lower values representing stable and adjustable weekly training load to players’ athletic performance.

### Players’ Match Performance

The Instat Index (Instat, Moscow, Russia) calculates a match performance indicator for each player. It is a unique parameter that provides an assessment of a player’s match performance based on the combination of 12 to 14 performance variables, with a higher numerical value indicating better performance [23]. It is created by an automatic algorithm that considers the player’s contribution to the team’s success and the significance of their actions. The rating is created automatically, and each parameter has a factor that changes depending on the number of actions and events in the match.

### Team Match Performance

All official matches that allowed one week of preparation were considered, and for the analysis, the number of points achieved at the end of the match according to the result was considered. That is, 3 points correspond to victory, 1 point to a draw and 0 to defeat.

### Statistical Analysis

Descriptive statistics including means, range values and standard deviation, as well as bivariate correlations, were calculated for variables under analysis. In addition, a path analysis model via the maximum likelihood (ML) estimator method in AMOS 23.0 was performed to test the associations across studied variables [26]. Bootstrap resampling (1000 samples) via bias-corrected 95% confidence intervals (CI) was used to assess the significance of the direct and indirect effects. An effect is considered significant (at ≤ .05) if its 95% CI does not include zero [27, 28]. Effect sizes were evaluated as trivial (0–0.19), small (0.20–0.49), medium (0.50–0.79), and large (0.80 and greater) [29]. The model fit was assessed through the following traditional goodness-of-fit indexes: comparative fit index (CFI); Tucker-Lewis index (TLI); standardized root mean square residual and root mean square error of approximation (RMSEA) and its respective confidence interval (90%). For these indexes, the suggestions of several authors [26, 30, 31] were adopted: CFI and TLI ≥ .90; SRMR and RMSEA ≤ .08.

## RESULTS

### Preliminary Analysis

The inspection of the data revealed that missing values were less than .1% of the sample considered in the present study, and consequently the full information maximum likelihood estimation (FIML) was considered for analysis [32]. Additionally, no outliers (univariate and multivariate) were identified. Skewness and kurtosis values were within cut off values revealing no violation from univariate data distribution. Nevertheless, Mardia’s coefficient for multivariate kurtosis exceeds the recommended value (Byrne, 2016). Therefore, a BollenStine bootstrap (2000) was performed for further analysis. Finally, the collinearity diagnosis was checked via the variance inflation factor (VIF) and a tolerance test assuming values less than 10 for VIF and greater than .01 for tolerance test. Therefore, the results showed that both in VIF and tolerance tests scores were below 10 and above .1 respectively, ensuring the appropriate conditions to test the regression model [31].

### Descriptive Statistics

Table 1 presents the average values and respective standard deviation of the weekly training variables as well as the bivariate correlations between them. The results revealed that team match performance has a significant positive correlation with the player’s match performance (r = .55**) and in turn, the player’s match performance is significantly influenced by the CMJ-cv (r = -.33*).

All the other variables did not reveal significant correlations and can be found in Table 2.

**TABLE 2:** Descriptive statistics and bivariate correlations

Variables	M	SD	Range	1	2	3	4	5	6	7
1. PTP	1.45	1.42	0–3	1	-	-	-	-	-	-
2. sRPE	371.08	48.04	254.46–463.03	.07	1	-	-	-	-	-
3. TQR	15.70	.91	14.40–18.81	.10	-.10	1	-	-	-	-
4. WBs	67.45	4.72	59.33–77.56	.07	-.25	.17	1	-	-	-
5. CMJ-cv	.14	.06	.06–.34	.15	-.16	-.23	-.22	1	-	-
6. PMP	220.12	31.84	160.50–279.77	-.01	.09	.13	.12	-.33*	1	-
7. TMP	1.61	1.42	0–3	.23	.10	-.08	-.03	-.02	.55**	1

**Note.** M = Mean; SD = Standard deviation; PTP = Previous Team Performance; sRPE = Session Rated Perceived Exertion; WBs = Wellbeing Score; CMJ-cv = Countermovement jump – coefficient variation; PMP = Player’s Match Performance; TMP = Team Match Performance

* p < 0.05;

** p < 0.01.

### Path Analysis

**FIG. 1:**
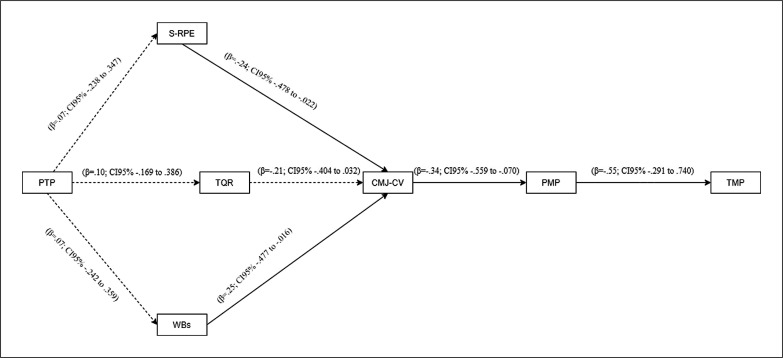
Performance explanatory model. Standardized direct effects of each path and sample Note: PTP = previous team performance; S-RPE = session rated perceived exertion; TQR = total quality recovery; WBs = well-being score; CMJ-cv = coefficient variation of countermovement jump; PMP = player match performance; TMP = team match performance.

The test of path analysis model included previous team performance, sRPE, TQR, WBs, CMJ-cv, players’ match performance and team match performance. The results show that the proposed model fit the data (χ^2^ = 14.71 (16); SRMR = .080; B-Sp = .473; RMSEA = .053 [90%CI = .000, .161]; TLI = .909; CFI = .929). Considering direct and indirect effects the proposed model explains 31% of match players’ and team performance. The standardized direct effects of each path and sample are displayed in Figure 1. The observed effects varied between trivial and medium. Regarding indirect effects, only a negative association between CMJ-cv and team match performance via player’s match performance (β = -.19; CI95% -.342 to -.049) was observed.

## DISCUSSION

The aim of this study was to investigate how the context defined by the match outcome of the previous match influences the weekly training load and well-being, and its contribution to explain players’ and team match performance of the following match. The proposed model for the training monitoring process explained 31% of team match performance. Bearing this in mind and knowing that success in futsal is the expression of a complex mix of technical, tactical, physical and mental factors [21], as well as all the factors that can affect the players’ performance or the success of the teams, the 31% that explains the team match performance appears to be a small, but considerable percentage for management by all the stakeholders in the training process.

The current main findings show no direct relationship between previous team performance and the following week’s training, and WBs variables. In addition, CMJ-cv proved to be a key variable to explain players’ and team match performance. To explain this finding, low values of CMJ-cv were related to higher individual and successful team match performance. Interestingly, higher values of weekly load and higher values of WBs were related to lower CMJ-cv.

Previous research in association football revealed a significant increase in perceived training load when a negative result (defeat or draw) occurred in previous matches [17]. Curiously, another study, with football teams, revealed greater total distance covered by players during training sessions after a win compared to a draw or defeat [18]. Also, with football it seems that when players lose a match, mood and stress are negatively affected, suggesting that the disappointment of losing a match could persist for several days [33]. Despite this, we find it interesting that regardless of the match result, futsal players manage to maintain their external load level [34].

In contrast to previous studies with football, and despite the differences between sports, our results did not reveal any significant relationship between the previous team performance and the weekly training variables (sRPE, TQR and WBs) considered. Thus, it seems that over the two seasons, internal weekly training load in futsal is not sensitive to previous team performance. These results, in comparison to previous results in football, may be explained by the fact that in futsal there are unlimited substitutions, and by the characteristics of training sessions mostly adopted in futsal, with the goal of maintaining the regularity in training load over weeks [7, 35]. Future studies should take into account the cumulative effect of loads over the weeks and be developed not only considering the match result, but also the subsequent match conditions and even the performance of players during such matches. Also, the analysis method should consider a more individualized approach that accounts for individual variability in time of play and other individual characteristics to further explain individuals’ variations in perception of the weekly training load [6].

The analysis of the weekly training load using subjective (sRPE, TQR and WBs) and objective (CMJ) measures [4, 14, 36] allows one to monitor variations in players’ physical performance and the state of readiness for competition.

While subjective measures contributed to evaluating the response of players to the session training load in relation to rest strategies and its influence on the general wellbeing, the objective evaluation of CMJ provided a reliable quantification of athletic performance and training-related fatigue of players [14, 36]. In line with that, our results revealed a significant negative relationship of sRPE and WBs with the CMJ-cv (the higher the sRPE and WBs values, the lower the weekly CMJ-cv).

Thus, despite a lack of correlation between sRPE and WBs, it seems that higher training load combined with higher wellbeing states could promote lower variation in CMJ and consequently ensures higher performance and readiness for competition [37–39]. These findings are in agreement with previous research which suggested that the monitoring process should account for variations in training load and in well-being responses [40]. In fact, based on our results, coaches cannot only think that the high sRPE is better. The range (˜254 to ˜463 AU) and the mean values (˜371 AU) of the daily sRPE variable were in line with the optimal range values for the daily training load for professional female futsal players [41]. Thus, the higher sRPE needs to ensure a balance between training load, recovery status, and WBs [4]. A correct balance of the training load is related to the greater probability for the success in the match outcome [19]. This result revealed that the weekly tracking of sRPE and WBs can give coaches the possibility to better manage training load and players’ fatigue state and performance readiness.

In this study, the analysis of weekly variations of CMJ (CMJ-cv) was revealed to be a key factor to synthesize balance in weekly training load with implications for players’ readiness to compete and to achieve higher individual performances and collective results. In opposition to previous research, in which external and internal load revealed trivial relationships with match statistics [9], the use of CMJ-cv seems to have clearly captured players’ readiness for performance.

In line with our results, lower values of CMJ-cv promoted higher players’ match performance. This is an interesting finding with clear implications for coaches’ intervention. This major finding suggests that correct variation of the training load, recovery status, and WBs during the microcycle [4, 36] allows players to maintain their level of neuromuscular performance and consequently to achieve high performance results in the match.

One of the limitations of the study is the fact that despite having a database from two full seasons, it only considered one team as a sample, which obviously limits the generalizability of the findings. Longitudinal, multi-team studies are therefore required to make more reliable inferences on the effects of individual factors on futsal performance.

## CONCLUSIONS

Contextual factors (previous team performance) seem to have no significant influence on monitoring the weekly training programme in futsal. The monitoring system defined by the analysis of the training load (as measured by session perceived exertion, sRPE), recovery status (TQR), players’ well-being (WBs) and neuromuscular performance (CMJ-cv) was fundamental to understand players’ readiness for competition with implications for their match performance and consequently for the final team result (team match performance). The methodologies we used could be applied to other similar sports to provide novel insights about performance.
